# Prospective Validation of the Decalogue, a Set of Doctor-Patient Communication Recommendations to Improve Patient Illness Experience and Mood States within a Hospital Cardiologic Ambulatory Setting

**DOI:** 10.1155/2017/2792131

**Published:** 2017-11-21

**Authors:** Piercarlo Ballo, Massimo Milli, Carly Slater, Fabrizio Bandini, Federico Trentanove, Giulia Comper, Alfredo Zuppiroli, Stefania Polvani

**Affiliations:** ^1^Cardiology Unit, S. Maria Annunziata Hospital, Florence, Italy; ^2^Cardiology Unit, S. Maria Nuova Hospital, Florence, Italy; ^3^Narrative Medicine Laboratory, Local Health Authority, Florence, Italy; ^4^Cardiology Service, Mugello Hospital, Borgo San Lorenzo, Florence, Italy; ^5^Regional Health Agency of Tuscany, Florence, Italy

## Abstract

Strategies to improve doctor-patient communication may have a beneficial impact on patient's illness experience and mood, with potential favorable clinical effects. We prospectively tested the psychometric and clinical validity of the Decalogue, a tool utilizing 10 communication recommendations for patients and physicians. The Decalogue was administered to 100 consecutive patients referred for a cardiologic consultation, whereas 49 patients served as controls. The POMS-2 questionnaire was used to measure the total mood disturbance at the end of the consultation. Structural equation modeling showed high internal consistency (Cronbach alpha 0.93), good test-retest reproducibility, and high validity of the psychometric construct (all > 0.80), suggesting a positive effect on patients' illness experience. The total mood disturbance was lower in the patients exposed to the Decalogue as compared to the controls (1.4 ± 12.1 versus 14.8 ± 27.6, *p* = 0.0010). In an additional questionnaire, patients in the Decalogue group showed a trend towards a better understanding of their state of health (*p* = 0.07). In a cardiologic ambulatory setting, the Decalogue shows good validity and reliability as a tool to improve patients' illness experience and could have a favorable impact on mood states. These effects might potentially improve patient engagement in care and adherence to therapy, as well as clinical outcome.

## 1. Introduction

During the past few decades, the role of strategies aimed at improving doctor-patient communication has progressively gained importance [1]. By focusing how patients' and physicians' psychological and emotional states and moods unfold in time in the context of their relationship, these strategies intend to shed light on the singular significance of the individual experience by improving empathy, trust, and collaboration [2]. In addition, by favoring the patient's participation and engagement in clinical choices, these approaches may allow for additional benefits related to the patient's sense of agency and self-management in care, compliance to therapy, and follow-up treatment, as well as improved clinical outcomes [3–5]. In this regard, we recently proposed the clinical use of the Decalogue [6], a tool including simple communication recommendations for patients and physicians to facilitate the different phases of the care-giving and care-receiving relationship. The Decalogue is a component of NaMe-3, a project endorsed by the Local Health Authority of Florence, Italy, that endeavours to spread the culture of patient-centered medicine, integrating strategies to improve the doctor-patient communication in clinical practice [7].

This study was conceived to test the psychometrical construct validity and the reliability of the Decalogue as a tool to improve the patient's illness experience and to investigate its clinical impact on mood states and on the perception of understanding one's own state of health in a population of outpatients prospectively enrolled in a cardiologic ambulatory setting. For the purpose of this study, we a priori decided to use structural equation modeling (SEM) to assess a psychometric construct including predefined latent variables related to the perception of the quality of the patient-physician communication process and the resulting change in the patient's illness experience. From a clinical point of view, we hypothesized that an easy-to-use tool based on simple communication rules might favor the patient-doctor relationship, leading to beneficial effects on patient's mood and perceived comprehension of the state of health.

## 2. Material and Methods

### 2.1. Study Population and Design

**Figure 1:**
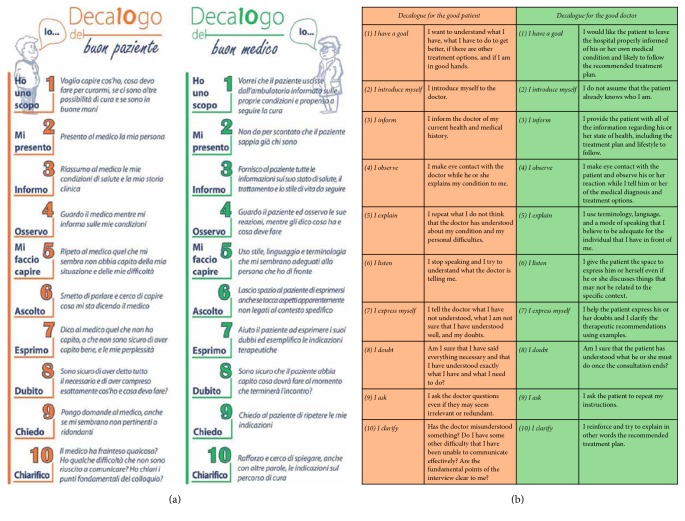
Main structure of the Decalogue. (a) The Decalogue flyer, printed in Italian language, used for this study. (b) English translation of the ten recommendations for the patient and the doctor.

The study population of this prospective, quasi-experimental study consisted of patients attending clinical cardiology consultations at two different Cardiology Units of the Local Health Authority of Florence, Italy, over a six-week enrolment period. Exclusion criteria were referral for instrumental examination without a clinical visitation, significant cognitive impairment, and refusal to participate in the study. Among a total of 102 patients who met the selection criteria during the enrolment period, 100 (98.0%) gave their consent for participation in the study and entered the study population. Multiple in-hospital meetings were held immediately preceding the start of the study in order to present the Decalogue to all physicians expected to be involved in the consultations. All patients were given detailed information about the study protocol the day of their scheduled examination. After study participants provided informed consent, each participant was provided with a copy of the Decalogue and was instructed to read it carefully before entering the examination room. The Decalogue is a set of 10 step-by-step communication recommendations used for facilitating communication between patients and physicians (Figure 1). These recommendations are subdivided into three phases, the initiation, development, and conclusion of the doctor-patient communication process, and invite both patients and physicians to focus on fundamental aspects of their relationship. Each of the ten recommendations is designed to confront a specific issue that may arise in the doctor-patient relationship and is described by a short title supplemented by additional notes. These notes highlight the significance of each issue, providing suggestions to confront it and aiming to contextualize that specific issue within the doctor-patient communication process as a whole. At the end of the clinical encounter, patients were moved to another examination room and were asked to answer to three different questionnaires. One questionnaire tested the validity of the Decalogue from a methodological standpoint and two others evaluated its clinical utility. Patients were also asked to provide an estimate of the time needed to read the Decalogue. A control group of 49 patients did not receive the Decalogue and were visited by physicians who had not participated in the in-hospital meetings presenting the Decalogue. To minimize the risk of selection bias, the controls were randomly enrolled among subjects referred for a cardiologic visitation in a third Cardiology Unit of the same Local Health Authority, during the same study period and irrespective of the referral reason or the results of the visitation. The controls were moved to another ambulatory room at the end of the clinical visitation and asked to answer the two questionnaires used for the clinical validation.

Data for the following variables were collected from each participant: age, gender, education level (elementary school, middle school, high school, and degree), socioeconomic status (low, intermediate, or high), main clinical reason for referral, current medications and doses, and history of depression. All data were collected in a predefined form, specifically designed before the beginning of the study for both patients and controls. To minimize the risk of information bias, we checked the number of cardiologic visitations performed in the year before the enrolment, as a measure of the intensity of medical surveillance, and consider it in multivariable analyses. Informed written consent was obtained from all participants. The study protocol complied with the Declaration of Helsinki and was approved (prot. 2/13 of June 6, 2013) by the local Ethics Committee.

### 2.2. Methodological Validation

*Construct Validity*. The first questionnaire was designed to test the validity of the psychometric construct underlying the Decalogue. Patients were asked to complete a validation questionnaire that resembled the main structure of the Decalogue and consisted of a series of 10 questions. Patients were asked to provide a score on a 7-level Likert-type scale for each item and to determine how much that specific item had been adequately achieved during the clinical visitation. The scales used for this analysis were both visual and numerical ones, as they included smiley symbols to favor the comprehension of the meaning of the numbers from 1 to 7. Results were then assessed by utilizing SEM with confirmatory factor analysis, a technique often used in psychometric studies that tests the validity of a given instrument by exploring the relationships between observed variables (that can be directly measured) and latent variables (that can only be inferred by observed variables through predictive models) [8, 9]. In SEM, observed variables can act as reflective measures (simple indicators of the corresponding latent variable) or formative variables (factors that potentially cause the latent variable). Analysis using SEM starts from a predefined path diagram that shows the expected relationships between variables where latent variables are shown as circular or oval boxes, observed variables are as rectangles, and arrows are used to designate the associations tested by the model. Once the path diagram is completed, specific path coefficients for each arrow can be calculated to determine the strength of the corresponding association and to obtain indexes for the applicability of the model.

**Figure 2:**
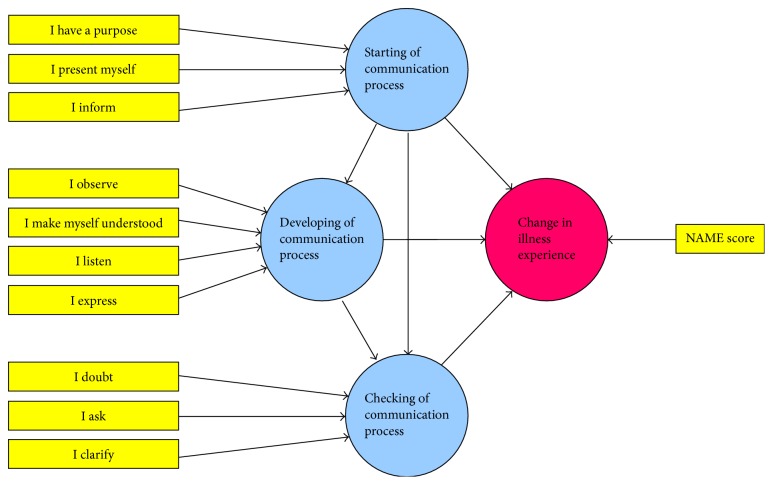
Graphical representation of the a priori structural equation model hypothesized to validate the psychometric construct. Latent variables are shown as circular boxes, while observed variables are indicated by rectangles.

For the purpose of this study, a psychometric construct was formulated in which a set of formative observed variables included the 10 specific item scores of the validation questionnaire. The model included four latent variables: the quality of the three main sequential phases of the patient-physician communication process (initiation, development, and conclusion) and the resulting change in the patient's illness experience (Figure 2). To obtain a measurable factor that could quantify this change in the illness experience, under the hypothesis that a better perception of understanding and increased awareness of the patient's own state of health could be associated with an improvement in the subjective illness experience, a second questionnaire based on four items was then introduced. These four items, diagNosis, Agents, lifestyle Modification, and lifE, together giving the abbreviation, NAME, served to evaluate the patient's understanding of the medical diagnosis, treatment goals and potential outcomes, and possible lifestyle changes and consequences. The questionnaire included the following 4 questions:

1. Did I understand which disease do I have?
2. Did I understand why I have to take my medications?
3. Did I understand how to change my lifestyle?
4. Did I understand how my disease will affect my life?

Patients were asked to provide a score on a 7-level Likert-type scale for each of the four items, and the total NAME score was calculated. This score was inserted into the construct model as a determinant of the changes in the patient's illness experience. To account for confounding effects, further analyses were performed by testing additional determinants, age, gender, education level in four levels, socioeconomic status in three levels, main clinical reason for referral, current medications and dosages, and history of depression, and assessing the influence of these determinants on the construct model.

*Reliability*. The reliability of the Decalogue was assessed using two different approaches aimed at estimating internal consistency and test-retest reproducibility. First, the split-half method was applied by separately considering odd- and even-numbered items, determining the correlation between these two split halves, and then stepping up to the full questionnaire with the Spearman–Brown prediction formula. This method measures the extent to which all items of the instrument contribute equally to the variables being measured and produces a coefficient that expresses the internal consistency of the instrument, with values approaching 1 indicating optimal performance. Internal consistency was also tested using Cronbach's alpha, a measurement that represents the mean of all possible split-half coefficients and provides an alternative estimate of the overall internal consistency of the Decalogue across items. The questionnaire was also administered to a subset of 15 subjects at least one week following the index clinical examination in order to assess the reproducibility of the Decalogue. Variability coefficients and intraclass correlation coefficients were calculated and used as measures of test-retest reproducibility.

### 2.3. Clinical Validation

*Assessing Effects on Mood States in Patients*. To investigate the effect of the Decalogue on patient mood states, the Profile of Mood States-2 (POMS-2) questionnaire was administered to both patients and controls. The POMS-2 is a multidimensional, comprehensive questionnaire commonly used to assess transient and fluctuating mood states and enduring states of affect [10]. This instrument includes 65 items and explores the patterns of mood states over six different scale scores, related to anger-hostility, confusion-bewilderment, depression-dejection, fatigue-inertia, tension-anxiety, and vigor-activity. The questionnaire provides specific scores for each of the six mood classes and a total score expressing the overall patient mood state (total mood disturbance score), with lower or more negative values indicating a more disturbed mood state.

*Effects on Patient Comprehension of His/Her Condition*. To investigate the effects of the Decalogue on the patient comprehension of the medical diagnosis, recommended treatment, and potential lifestyle changes and consequences, the NAME questionnaire used for the methodologic validation analysis was also administered to the control group.

### 2.4. Sample Size Calculations

In order to determine the sample size for the study, a significant difference in the total mood disturbance scores between the Decalogue test group and the control group was hypothesized. Assuming an overall SD of 15 for the score in the overall population and a 2 : 1 patient-control ratio, a total sample size of 144 subjects (96 in the Decalogue group and 48 in the control group) would have allowed detecting a score difference between groups of 7.5, corresponding to an effect size of 0.5 (i.e., medium), with 80% power at a 0.05 significance level. The study period needed to achieve these sample sizes was a priori estimated between six and eight weeks. Enrolment was stopped during the first six weeks of the study when adequate sample sizes had been obtained.

### 2.5. Statistical Analysis

Data were expressed as mean ± SD or median [IQR]. Normality was assessed by the Shapiro-Wilk test. Differences between patients and controls were explored by the Student *t*-test for independent samples, whereas the Mann–Whitney *U* test was used for nonparametric variables. Categorical variables were compared using the chi-square test or the Fisher exact test, as appropriate. Multivariable regression was used to check the independent effect of the Decalogue on clinical outcomes after adjustment to confounders. For the methodological validation, SEM was initially performed by building a predefined path diagram including 4 latent variables and 11 formative observed variables (Figure 2). To account for the effects of confounders, additional variables that could potentially affect the patient illness experience were successively inserted as formative measurable variables and tested. The fitting of the multidimensional three-factor model was compared to more parsimonious nested models (unidimensional and bidimensional) by assessing the model chi-square difference, taking into account the different degrees of freedom. Path coefficients and model fitting indexes, expressed as *R*^2^ values, were calculated using the partial least squares algorithm that utilizes a sequence of regressions in terms of weight vectors so that vectors obtained at convergence satisfy fixed point equations [11, 12]. For this analysis, a path weighting scheme with a maximum number of iterations set to 1000, initial outer weights set to +1, and a stop criterion of <10^−7^ were utilized, considering that a change in the outer weights between two consecutive iterations smaller than this value would imply acceptable convergence. In all analyses, the algorithm was not stopped when the maximum number of iterations was reached, but rather only at the stop criterion. Bootstrapping was used to test the significance of formative indicators' outer weights. Among confounding variables, only those achieving significance were kept in the final path diagram. To test for convergent validity, a redundancy analysis was performed separately for each latent variable. The NAME score was considered as a reflective indicator for the initiation, development, and conclusion of the doctor-patient communication process, while all 10 indicators of the items in the Decalogue were regarded as formative variables for the patient illness experience. Collinearity diagnostics were also performed to ensure model stability, and values of variance inflation factor >5 were used to indicate collinearity problems. In all analyses, quantitative variables were all considered as continuous variables without any grouping, to minimize the loss of information. We also a priori decided to use missing data analysis procedures only in case of variables with >5% of missing data. The significance level was set at 0.05, and all tests were two-tailed. SEM was performed using SmartPLS version 2.0, Hamburg, Germany. All remaining analyses were performed using SPSS for Windows release 13.0 (Statistical Packages for Social Sciences Inc., Chicago, IL).

## 3. Results

### 3.1. Main Characteristics

The two study groups showed no significant differences in the main variables (Table 1). In both groups, the majority of patients were of relatively low levels of education, and more than two-thirds of the subjects were of a low socioeconomic status. Coronary artery disease, heart failure, arrhythmias, and hypertension were the most common clinical reasons for referral in the overall population of patients and control subjects. Most individuals were also on multiple medications for the aforementioned conditions. The prevalence of depression was low in both groups. The estimated time needed to read the Decalogue was <5 minutes in 43 patients and <10 minutes in 92 patients.

### 3.2. Methodological Validation

**Figure 3:**
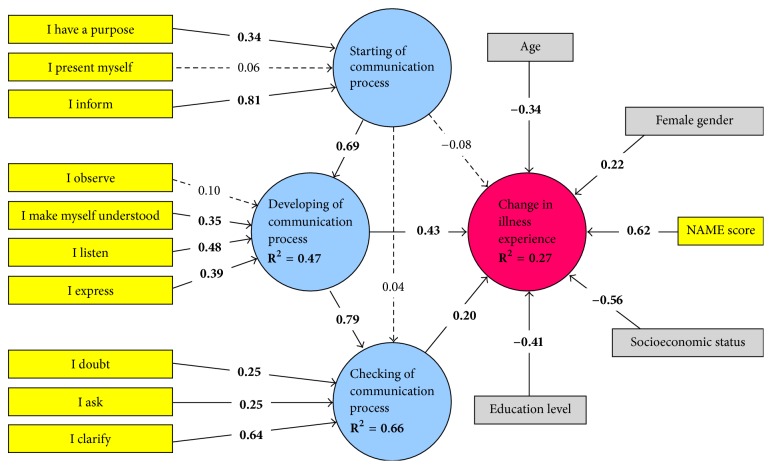
Results of structural equation modeling analysis. Path coefficients for both formative variables included in the a priori model (in yellow) and confounding variables (in grey) are reported. Model fitting indexes, expressed as *R*^2^ values, are also shown. Dotted arrows denote nonsignificant associations.

*Construct Validity*. The final psychometric construct of the Decalogue, as obtained by SEM, is illustrated in Figure 3. Most indicators related to the 10 items of the Decalogue had a significant effect on the quality of the three phases of patient-physician communication process (initiation, development, and conclusion). With regard to the latent variables, the initiation of the communication process had a significant impact on successive communication development, which in turn strongly determined the conclusion phase.

The quality of communication during the development and conclusion phases of the process, but not during initiation phase, showed a significant positive effect on the patient's illness experience. The following confounding variables, younger age, female gender, low level of education, and low socioeconomic status, were found to positively affect the change in the patient's illness experience. All latent variables showed acceptable convergent validity, with all coefficients >0.80. The model fitted better than 2-dimensional or unidimensional models (*p* < 0.001 for chi-square difference in all model comparisons). The overall model explained 47% of the variability in the quality of communication development, 66% of that in the quality of communication conclusion, and 27% of the resulting change in the patient's illness experience.

*Reliability*. The split-half method provided a coefficient of 0.87 and the Cronbach alpha was 0.93, indicating optimal internal consistency. The Decalogue also presented high test-retest reproducibility, as shown by a variability coefficient of 6.5% and an intraclass correlation coefficient of 0.92.

### 3.3. Clinical Validation

**Figure 4:**
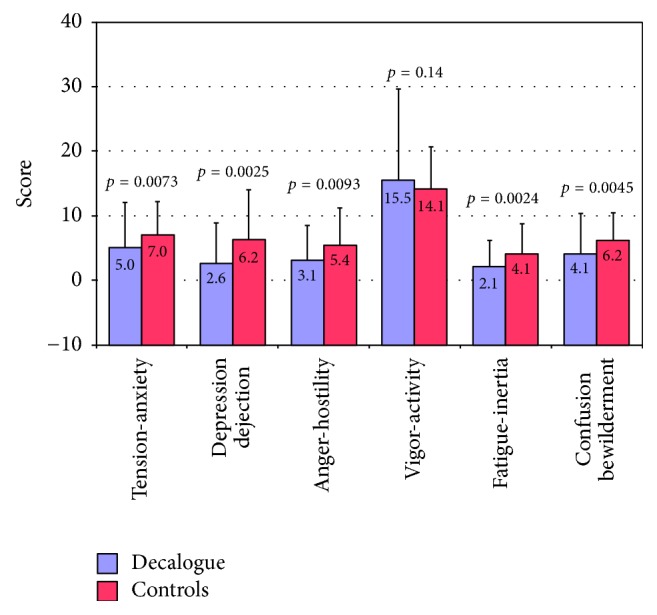
Differences in the specific mood disturbance scores for the single classes of the POMS-2 questionnaire between the Decalogue group and the controls.

*Effects on Patient Mood States*. The total mood disturbance score was lower in the Decalogue group as compared to the control group (1.4 ± 12.1 versus 14.8 ± 27.6, *p* = 0.0010). This difference remained significant after adjustment to age, gender, education level, socioeconomic status, main clinical reason for referral, current medications, history of depression, and number of cardiologic visitations during the last year (*p* = 0.0025). Specific mood disturbance scores were all consistently lower in the Decalogue group than in the control group for nearly all single classes of POMS-2 questionnaire (Figure 4).

**Figure 5:**
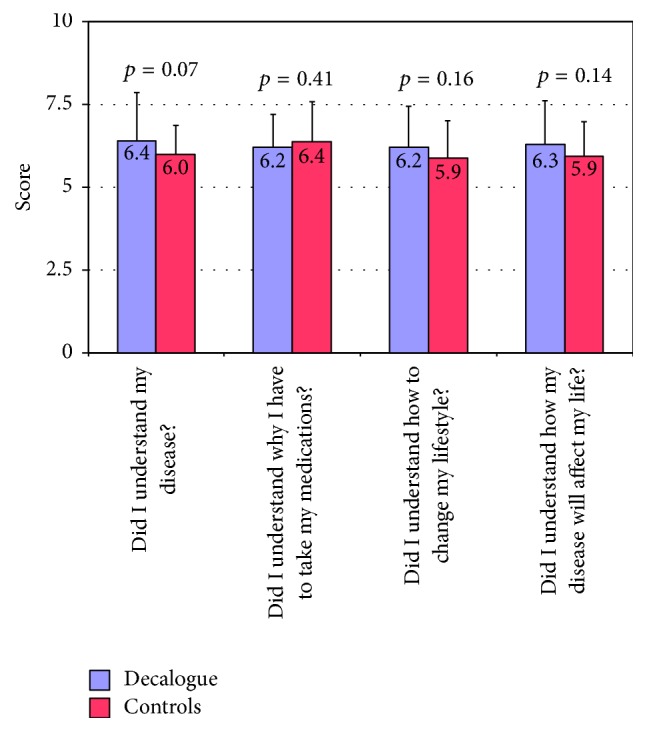
Differences in the specific scores for the single items of the NAME questionnaire between the Decalogue group and the controls.

*Effects on Patient Understanding of State of Health*. The total NAME score did not differ between the Decalogue group and the control group (25.1 ± 4.2 versus 24.1 ± 3.9, *p* = 0.17). Specific scores did not differ between the two groups, although there was a borderline trend towards a better understanding of one's own state of health in the Decalogue group as compared to the controls (Figure 5).

## 4. Discussion

### 4.1. Main Findings

It is widely recognized that psychological issues play an important role in the clinical management of patients with cardiovascular diseases [13, 14]. Negative affective states are associated with increased cardiovascular risk, and interventions aimed at reducing psychosocial symptoms have been shown to provide a clinical benefit in primary and secondary cardiovascular prevention [15–19]. In this regard, strategies to improve doctor-patient communication may also favor clinical outcome as a result of the potentially beneficial effects on the patient illness experience, mood stabilization, and the patient's understanding of his or her state of health, which may consequently favor better engagement and sense of agency in care, greater confidence and trust in the physician, and an improved compliance to therapy and follow-up treatment [20, 21]. In this study, the Decalogue, a set of simple doctor-patient communication recommendations, was tested for its validity and reliability as a tool to improve the patient illness experience and assessed for its clinical effects on patient mood states and on the patient's understanding of his or her own state of health. In a population of outpatients prospectively enrolled in a cardiologic ambulatory setting, it was found that (1) the Decalogue showed sufficiently good psychometrical validity and reliability; (2) when given to patients before a cardiologic consultation, it seems to yield some improvement in patient's mood states and in the perceived comprehension of his or her state of health.

### 4.2. Psychometric Validity and Reliability of the Decalogue

Defining a consistent psychometric construct is a primary issue for the methodological validation of any instrument aimed at interacting with patients' psychosocial variables [22]. Due to the fact that this analysis usually deals with variables that cannot be measured directly (i.e., latent variables), it is commonly performed by predefining a construct and testing its validity by SEM. SEM is a technique that tests the validity of complex sets of relationships between observed and latent variables [23] and that has been previously used for the validation of several tools, instruments, and questionnaires [24–27]. In this study, a construct linking the different items of the Decalogue to the quality of the main phases of patient-physician communication process, initiation, development, and conclusion, was predefined, under the assumption that changes in the quality of these phases could change the patient's illness experience [28] The association of the illness experience, defined as an individual's unique response to his or her medical condition, with clinically relevant variables, such as adherence to medications, engagement and sense of agency in care in care, and compliance to follow-up treatment, was established [29–31]. The results of SEM analysis seem to indicate a good validity of this construct. The quality of the three communication phases showed a significant association with the items of the Decalogue and in turn demonstrated a positive impact on the patient illness experience, explaining 27% of its variability. An acceptable reliability of the Decalogue construct was also observed, as indicated by both internal consistency indexes and test-retest reproducibility analysis. These findings suggest that, by calling the patient's attention to a few communication recommendations before a cardiologic examination, the Decalogue might, in fact, improve the quality of doctor-patient communication and potentially result in favorable effects on the patient's illness experience.

It was also found that the effect on the patient illness experience was positively associated with female gender and negatively associated with age, socioeconomic status, and education level. The effects of age and gender differences on the trajectories of individual illness experiences have been widely recognized, with most analyses showing a greater reactivity of women and younger subjects [32–37]. Discrepancies in the illness experience related to socioeconomic, racial, and cultural factors were also reported in a number of pathophysiological conditions [38–43]. With this in mind, the findings of this study suggest that the beneficial impact of the Decalogue on the patient's illness experience may be particularly evident in younger individuals, women, and subjects coming from low socioeconomic classes and low levels of education.

### 4.3. Clinical Utility of the Decalogue and Study Limitations

At the end of the cardiologic visitation, patients who had received the Decalogue showed a lower total mood disturbance score as compared to control subjects of the same age, gender distribution, socioeconomic class, and education level. This effect was consistently observed across nearly all specific mood classes, including anger-hostility, confusion-bewilderment, depression-dejection, fatigue-inertia, and tension-anxiety. In addition, patients who had received the Decalogue showed a trend towards a better understanding of the medical diagnosis in comparison with the control group patients. The association of negative moods such as anxiety, anger, hostility, depression, job burnout, and perceived stress with adverse cardiovascular outcome was demonstrated in a number of studies in both healthy and diseased populations [44–50] and is likely to depend on a complex interaction between genetic, biochemical, neurohormonal, and autonomic factors [51–54]. Part of this association is mediated by poor adherence to medical treatment and coping with negative mood states by engaging in unhealthy behaviours such as smoking, overeating, or drinking [55, 56]. Regardless of the mechanisms, the finding of an improved mood state and a tendency towards a better perception of understanding the state of health in the Decalogue group might be of clinical interest because of the potentially beneficial impacts on sense of self-empowerment and agency in care, quality of life, therapeutic compliance, and follow-up treatment. While these findings should be interpreted with caution given that long-term follow-up data are not currently available, the effects of the Decalogue demonstrated in this study suggest that the Decalogue may improve the psychological well-being of the patient, a variable of clinical importance because of its expected favorable impact on clinical outcome [57].

Several limitations should be considered in this study. Although the control group was comparable to the Decalogue group with respect to the main variables, a major issue is that the study design did not include a predefined formal matching procedure. Since we only measured the patient's perception of understanding his state of health, nothing can be concluded about the true level of state of health comprehension. Similar considerations can be made for the quality of the communication process, for which only the perceived quality was measured. This study was specifically designed to explore the effects of the Decalogue on outpatients referred to hospital cardiologic ambulatory facilities, and thus the findings cannot be generalized outside of this context. The sample size did not allow us to investigate the effects of the Decalogue in subsets of patients with different cardiovascular syndromes. The possibility of statistical artefacts due to the design of the study cannot be excluded. Also, it should be noted that some of the relations between confounding variables and change in the patient's illness experience were expected, taking into account the composition of the study population. Lastly, further studies are warranted to explore the prognostic impact of the Decalogue, particularly on the risk of hard clinical events.

## 5. Conclusions

In conclusion, in a population of outpatients undergoing a cardiologic consultation in a hospital ambulatory setting, the Decalogue could have sufficient validity and reliability as a tool for the improvement of the patient's illness experience. It might also provide some beneficial effects on patient's mood states and the perceived understanding of his or her own state of health. These effects could potentially favor clinical outcomes.

## Figures and Tables

**Table 1:** Comparison of general characteristics between the two study groups. CAD = coronary artery disease.

	Decalogue group (*n* = 100)	Control group (*n* = 48)	*p* value
Age (years)	67.5 ± 15.9	70.6 ± 13.5	0.22
Female gender (*n*)	46 (46.0%)	19 (38.8%)	0.51
Education level (*n*)^*∗*^			0.74
Elementary school	27 (27.8%)	15 (31.3%)	
Middle school	30 (30.9%)	13 (27.1%)	
High school	29 (29.9%)	12 (25.0%)	
Degree	11 (11.3%)	8 (16.7%)	
Socioeconomic status (*n*)^*∗*^			0.81
Low	71 (73.2%)	33 (68.8%)	
Intermediate	16 (16.5%)	10 (20.8%)	
High	10 (10.3%)	5 (10.4%)	
Main reason for referral (*n*)			0.88
CAD	37 (38.0%)	15 (31.3%)	
Heart failure	30 (28.0%)	17 (35.4%)	
Arrhythmias	17 (18.0%)	7 (14.6%)	
Hypertension	7 (7.0%)	5 (10.4%)	
Other	9 (9.0%)	4 (8.3%)	
Number of medications in current therapy (*n*)	5 [3–7]	5 [4–7]	0.56
Number of daily medication assumptions (*n*)	6 [3–9]	6 [4–9]	0.67
Depression (*n*)	7 (7.0%)	1 (2.3%)	0.28

^*∗*^Percentages calculated on a total sample of 97 patients in the Decalogue group, due to missing data in 3 subjects.
